# Enhancing Collaborations between our Age-Friendly Community and University Initiatives

**DOI:** 10.1093/geroni/igab046.376

**Published:** 2021-12-17

**Authors:** Wendy Rogers, Brian Pastor

**Affiliations:** University of Illinois Urbana-Champaign, Champaign, Illinois, United States

## Abstract

Community involvement and synergistic partnerships are key to fostering a holistic approach to programming and outreach that assess and meet the needs of the older adults in our communities. The University of Illinois Urbana-Champaign has created an Age-Friendly network to facilitate these partnerships featuring our designation of Age-Friendly City and Age-Friendly University as well as partnerships with our state and local governments, community aging services providers, and continuing care retirement communities. Through these partnerships, we have identified the landscape of the community, assessed the unique needs older adults, and identified novel solutions. We will discuss our plans for activities that will empower older adults in our community and at our university by promotion connection, collaboration, and inclusion.

